# GenPADS: Reinforcing politeness in an end-to-end dialogue system

**DOI:** 10.1371/journal.pone.0278323

**Published:** 2023-01-06

**Authors:** Kshitij Mishra, Mauajama Firdaus, Asif Ekbal

**Affiliations:** Department of Computer Science and Engineering, Indian Institute of Technology Patna, Bihta, Bihar, India; Sreenidhi Institute of Science and Technology, INDIA

## Abstract

In a task-oriented dialogue setting, user’s mood and demands can change in an ongoing dialogue, which may lead to a non-informative conversation or may result in conversation drop-off. To rectify such scenarios, a conversational agent should be able to learn the user’s behaviour online, and form informative, empathetic and interactive responses. To incorporate these three aspects, we propose a novel end-to-end dialogue system **GenPADS**. First, we build and train two models, *viz.* a politeness classifier to extract polite information present in user’s and agent’s utterances and a generation model (G) to generate varying but semantically correct responses. We then incorporate both of these models in a reinforcement learning (RL) setting using two different politeness oriented reward algorithms to adapt and generate polite responses. To train our politeness classifier, we annotate recently released Taskmaster dataset into four fine-grained classes depicting politeness and impoliteness. Further, to train our generator model, we prepare a **GenDD** dataset using the same Taskmaster dataset. Lastly, we train **GenPADS** and perform automatic and human evaluation by building seven different user simulators. Detailed analysis reveals that **GenPADS** performs better than the two considered baselines,*viz.* a transformer based seq2seq generator model for user’s and agent’s utterance and a retrieval based politeness adaptive dialogue system (PADS).

## 1 Introduction

Conversational systems are ubiquitous with their widespread applications in our everyday lives. Advancements in artificial intelligence (AI) has made it possible for the conversational agents to assist us with our daily chores such as booking flights, reserving tables, scheduling movies, etc. With ongoing growth in the field of natural language processing (NLP), it is crucial to make these agents human-like. Lately, prior research has focused on making the conversational agent empathetic [1–4], personalized [5–7] and courteous [8, 9]. One of the long-standing objectives of AI is to make the conversational agents behave like humans. To ensure hearer engagement, with *content-aspect*, it is seen that a human-human conversation focus on *meta-communicative* aspect of language also which ensures *‘How the content is expressed?’*. Hence, to achieve an interactive and engaging conversation, it is crucial for a dialogue agent to inculcate human like manners in these agents.

**Fig 1:**
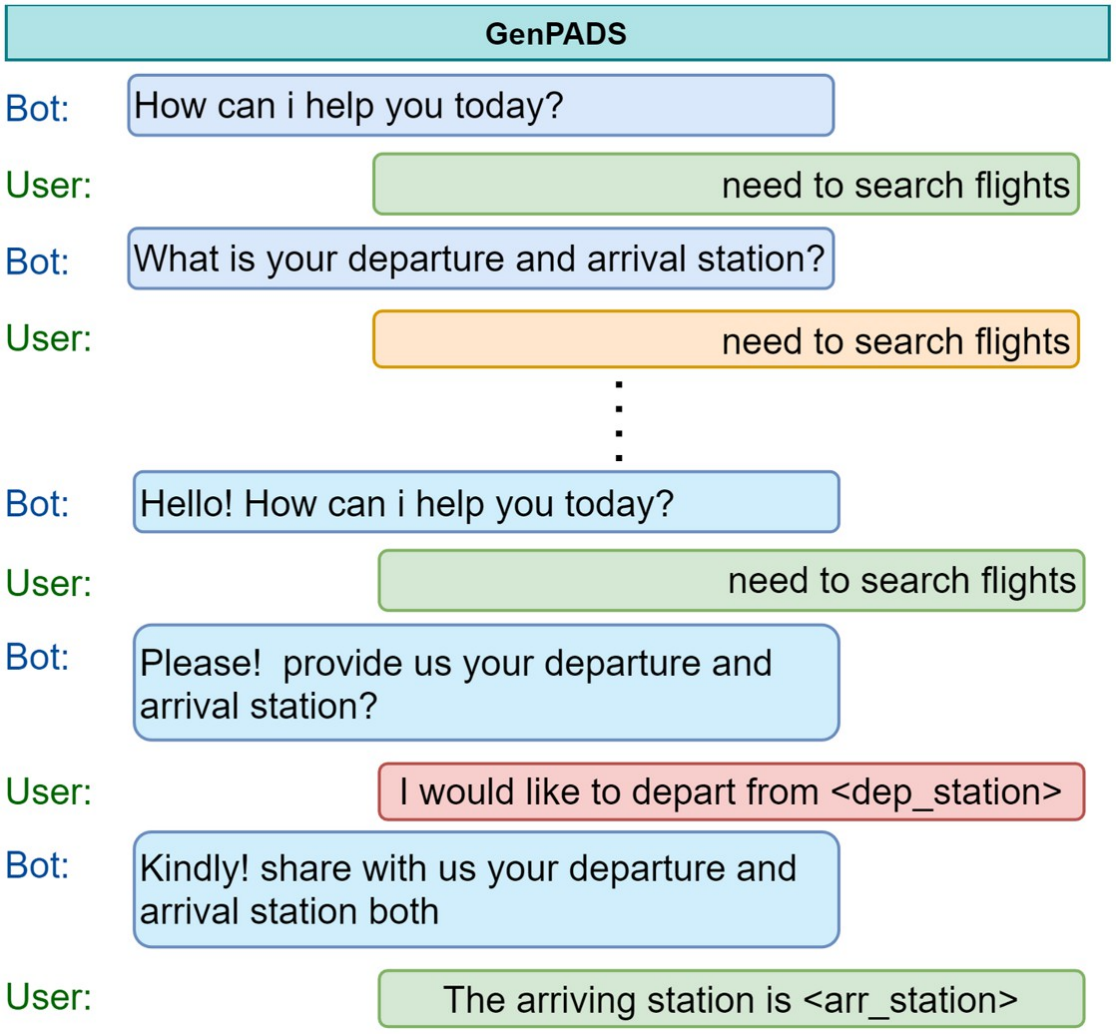
An example of generative politeness adaptive dialogue system. Light orange (impolite or noisy) and light red (partial information) utterance boxes depict the user’s dissatisfaction with the ongoing dialogue. *GenPADS* adapts the dialogue system towards generating polite and diverse responses as per user’s and agent’s politeness feedback.

A goal-oriented dialogue system aims to address user queries and assist them with their everyday tasks. To ensure customer satisfaction and to increase retention it is essential for the agents to appropriately empathize, appreciate, apologize, assure and greet the customers in the best possible way. Incorporating politeness in conversations can make hostile discussions more smoother and interactive for both the agent and the end users. Motivated with real-life scenarios where on dissatisfaction of the user, agents behave in a more cordial and polite manner with their customers, we propose the task of introducing politeness in dialogues systems in an end-to-end fashion. As shown in Fig 1, we provide an example for the Flight domain. Here, the first half is a normal conversation while the second part is a polite conversation where the agent adapt towards politeness incorporation in the generated responses on encountering user’s dissatisfaction. Further, with a generation module a diverse response without the loss of semantics of generated utterance can be achieved. Hence, by including politeness and a generation module, we can make the agent much more human-like and interactive while the former seems merely like a question-answer system.

As the dialogue manager decides “what to say” and the natural language generator decides “how to represent” the information to the user hence, it is important that the incorporation of politeness in dialogues takes place by modeling both of these modules simultaneously. For correct extraction of the information to provide informative responses infused with politeness requires the conversational agent to work in close coordination with the dialog manager and the generator module. Therefore, we design an end-to-end framework that manages the dialogue and generates the corresponding polite response making it one of the first works that performs two crucial tasks, *viz.* dialogue management and response generation in a dialogue system concurrently.

To build our system, first, we trained tranformer based politeness classifier (**PC**)—to receive polite feedbacks and a geneartion module (**G**)—to generate diverse, interactive, but semantically similar responses. Second, **PC** and **G** are incorporated in an RL based framework by designing an effective politeness based reward function. The proposed reward function takes politeness feedbacks given by **PC** in form of rewards. In case of negative rewards received, proposed RL based system adapt towards polite actions to satisfy the user.

The key contributions of our current work can be summarized as follows:

1. Annotated the Taskmaster dataset with fine-grained four politeness labels *viz.* impolite, somewhat_impolite, somewhat_polite and polite. We name this dataset as **PADD** which is used to train a transformer based politeness classifier **PC**.
2. Utilising Taskmaster dataset, prepared a sequence-to-sequence generation dialogue dataset (**GenDD**) to train the a generation module **G**.
3. Designed a politeness based reward function that controls the dialogue agent to generate polite responses on user dissatisfaction with the ongoing dialogue.
4. Proposed an end-to-end generative politeness adaptive dialogue system **GenPADS** in a reinforcement learning framework that performs dialogue management and response generation simultaneously to generate polite, diverse, and interactive responses.
5. Performed detailed experimental analysis of each component of our proposed system, **GenPADS**, to showcase that its strength with respect to both the automatic and human evaluation metrics compared to the baselines.

## 2 Related work

The other party in the dialogue has the inherent ability to directly injure the addressee’s face by acting in opposition to the addressee’s wants and desires [10]. Hence, to preserve each other’s faces during an interaction, participants must cooperate and maintain each other’s face [11]. These affronts to a person’s self-esteem can be mitigated by employing polite responses in interactions [12, 13]. According to the politeness theory [10], humans have a basic need for acceptance and approval, sometimes known as positive face. Based on this approach, we in this work propose that language elements, such as aggressive language, used to elicit the required response can be perceived as a threat to autonomy (i.e., negative face), hence, may affect the ongoing conversation and further engagement of the user negatively.

Politeness and its close relation with power dynamics and social interactions has been well documented in [10]. Formerly, there have been a few attempts to incorporate politeness in response generation [8, 9, 14, 15]. In [8], the authors proposed the task of transforming a generic customer care response into polite response utilizing reinforced pointer generator networks. While in [9], conversations were made polite without any parallel data employing reinforcement learning. In [14], the phrases *“please”* and *“could you”* signal on sentence heatmaps as examples of how a computational linguistic system can learn to be polite. Recently, the authors in [15] designed a tag and generate pipeline that identifies stylistic attributes and subsequently generates a sentence in the target style (polite) while preserving most of the source content.

Research on dialogues systems has recently focused on combining different modules in an end-to-end learning framework [16–20]. Reinforcement learning (RL) has also facilitated building dialog systems [21–23]. A Deep Recurrent Q-Networks (DRQN) was employed in [21] for building an end-to-end framework for task-oriented dialog systems. A deep RL framework for iterative dialog policy optimization in end-to-end task-oriented dialog systems was presented in [22]. Users’ feelings in the form of sentiments and emotions have been exploited in the past for building effective dialogue systems [24–28]. In [24] authors built a virtual bot named ‘Zara’ which tries to evince empathy using facial emotion recognition. The authors in [25] utilized the user sentiments obtained from multimodal sources in the end-to-end dialog framework to make the system user-adaptive and efficient. Similarly, in order to enhance the user experience and increase satisfaction, in [29], authors addresses the combined impact of sentiment and emotion while generating responses.

Our current work differs from the existing works on politeness as the existing works either focused upon identifying politeness or generating polite responses. While we design an end-to-end dialogue framework that utilizes the polite classifier to extract polite information from the utterances, controls the dialogue management, and uses a generator for producing polite responses in an RL-based polite adaptive dialogue system for creating an interactive and polite agent.

## 3 Dataset

In order to build the sub-components *viz.* politeness classifier (PC) and generation module (G) of our proposed system GenPADS, we prepare two datasets, *viz.* a politeness annotated dialogue dataset (PADD) for politeness classifier module and a seq2seq based generative dataset for generator module of our politeness adaptive dialogue system (GenPADD). We choose the recently released Taskmaster dataset [30] containing task oriented conversations between an agent and user in seven domains, *viz.* flights, food-ordering, hotels, movies, music, restaurant-search, and sports.

### 3.1 Politeness annotation

The existing politeness annotated dataset [31] is much more like a question answering dataset, containing requests requested to a addressee. This dataset is not suitable for modeling the variations of politeness in a dialogue system as we want to trace the user’s satisfaction with the ongoing dialogue as well as the agent’s way of responding. Therefore, to choose a common attribute which can define a user’s dissatisfaction with the dialogue, such as rudeness or plain responses, as well as the agent’s response quality, we decided on the ‘politeness’ attribute. To obtain the politeness aware dialogue dataset, we manually annotate Taskmaster-2 dataset with varying levels (i.e. fine-grained) of politeness. In a dialogue setting, defining only two politeness classes (i.e., polite or impolite) to train an end-to-end model may cause information loss. For example, in our case, we want our conversational agent to be polite as per the user’s polite feedback as there may be scenarios that user is engaged in the ongoing conversation being only somewhat polite/impolite to the user. Furthermore, there may be cases that a user may show dissatisfaction with agent’s somewhat_impolite response such as: Could you share with us your departing station?. Hence, the agent should be able to comprehend this and respond in a more polite manner such as: For further processing, could you please share with us your departing station? to adapt towards the different fine-grained level of politeness. Therefore, to prepare PADD, we define four fine-grained classes: 0—impolite (’What is your food order?’), 1—somewhat_impolite (Can you tell us your food order?), 2—somewhat_polite (Please, provide your food order details?), 3—polite (Could you please, share with us your food order details?).

We did not choose to use only polite keywords to identify the politeness level of an instance as it is possible that the instance can be polite/impolite even if there are no polite keywords based on context only. For example, *‘Do not worry, we are working on your problem’*—somewhat_polite or *‘It’s not too much out of your way, just a couple of bucks’*—somewhat_polite. Further, instead of only keywords, impoliteness/politeness heavily depends on other contextual words, i.e. a polite keyword can make a sentence impolite, or vice-versa, for example, *‘It’s impolite to not help you’*—polite; *‘It’s polite to not help you’*—impolite. Here, use of keywords polite and impolite are contradicting each other. Similarly, the same keyword can affect an utterance in different ways as per the different qualifiers or dependent words available. For example, *‘It is good to be of help’*—somewhat_polite; *‘It is not good to be of help’*—somewhat_impolite. Here, the use of qualifier *‘not’* is affecting the politeness of the keyword *‘good’*. Therefore, we need a classifier that can approximate a good function to map different utterances to different polite classes.

To have polite information feedbacks also, we required a politeness classifier as absence of polite keywords would have reflected negative quality of responses, which is not always the case as stated above through the examples. Lastly, to generate semantically similar but diverse responses incorporation of only different polite keywords in an agent’s response as per need, would result in diversity of polite keywords, but not in the response itself, which is our primary aim. For example, *‘Kindly, provide us details of your departing station?’* and *‘Could you please state your source station name?’* are two diverse but semantically similar polite responses.

**Fig 2:**
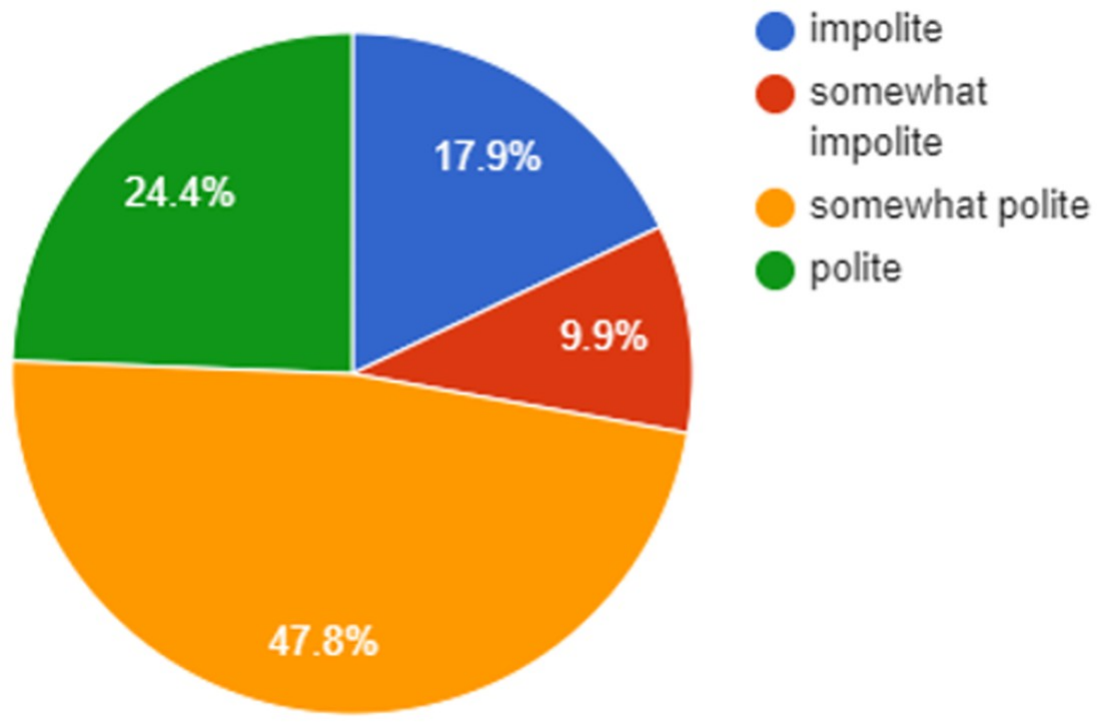
Distribution of different politeness classes in the Taskmaster dataset.

To annotate the utterances of the Taskmaster dataset, we employ crowd-workers from Amazon Mechanical Turk (AMT) that labels every utterance with the provided set of polite labels (i.e., polite, somewhat polite, somewhat impolite, and impolite) for the different domains. For labeling the utterances, the workers were asked to follow the instructions and guidelines provided for annotation. Some of the significant guidelines for annotation were as follows: (i) Each utterance was to be marked with one of the labels specified; (ii) To differentiate between polite/somewhat polite utterances the workers were asked to check for explicit usage of polite phrases in the utterance, such as thank you, you are most welcome, etc. (iii) Similarly, for the impolite/somewhat impolite utterance, we follow the explicit usage of the impolite markers to differentiate between these labels (iv) Annotators are asked to follow the guidelines given in [31] to annotate the utterances with correct politeness class, in case no polite phrases or markers are present in the instance. to counteract the different perception of annotators for an instance, maximum voted polite label is selected. A multi-rater Kappa [32] agreement ratio of approximately 80% was observed for the politeness annotation which can be considered as reliable. Distribution of annotated polite classes for the dataset is shown in Fig 2.

For response generation, we use the Taskmaster dataset having seven domains and build two sequence-to-sequence (seq2seq) generator models *viz.* Dialogue generator (DG) and GenPADS generation module (G). We prepare two variants of this data: DG-Dataset and G-Dataset, of which the former is used to train the DG and later is used to train the **G**. This dataset is named as generation dialogue dataset (GenDD).

To prepare both the datsets for generation, we first clean the available Taskmaster-2 dataset by dropping those utterances which are null or out of domain (Cleaned). Second, we remove non-informative utterances in each of the dialogue and merge consecutive user’s or agent’s utterance into a single utterance. Hence, the resultant dataset is present with alternative pairs of user’s and agent’s utterances in a dialogue. We name this dataset as DG-dataset and is use to train **DG** model, where user’s utterance is given as input and subsequent agent’s utterance as output. Further, we extract only agent’s utterances from this DG-dataset and perform forward-backward machine translation (*English* → *Chinese* → *English*) to generate two similar candidate responses. For each domain, randomly sampled 1k machine translated utterances are cross-verified manually and rest are kept same. Now, we consider these two candidate responses for each of the input agent’s utterance as target output to build and train our GenPADS generation module (G). We name this whole dataset consisting of both DG-Dataset and G-Dataset as generation dialogue dataset (GenDD). The statsistics of PADD and GenDD are shown in Table 1.

**Table 1:** PADD show the number of instances in the gold-standard polite annotated data. Cleaned shows the statistics of cleaned Taskmaster data; DG-Dataset and G-Dataset shows the statistics of dialogue dataset used to train the two generation models.

	PADD		GenDD	
Domain	PADD	Cleaned	DG-Dataset	G-Dataset
flights	63346	28961	16012	8006
food-ordering	13954	6555	4154	2077
hotels	63049	27299	12750	6375
movies	59758	25550	16674	8337
music	26913	12472	8204	4102
restaurant-search	66206	25708	14198	7099
sports	48581	26118	19080	9540

## 4 Proposed methodology: GenPADS

To build GenPADS, we model a classifier and a generator to predict the appropriate polite classes and generate the responses, respectively. Further, we also build a baseline dialogue generator model to compare with our GenPADS framework.

### 4.1 Politeness classifier

The politeness classifier is modelled based on the DistilBERT architecture [33], where a compact model (here, DistilBERT) is trained to reproduce the behaviour of a larger model (BERT) using knowledge distillation technique. For an input utterance *u*_1: *n*_ (*u*_*i*_ represents the *d*− dimensional word embedding vector), instead of training the classifier model over hard targets, DistilBERT transfers the knowledge from a larger teacher model to a compact student model with a cross-entropy over the soft targets. It can be formulated as given below:

$$\begin{array}{c} L=\sum_{i}t_{i}*\text{log}\left(s_{i}\right) \end{array}$$ (1)

where *t*_*i*_ denotes the probability estimated by the teacher and *s*_*i*_ denotes the probability estimated by the student. Further, to expose the classes as probability mass of distribution, a softmax with temperature is introduced:

$$\begin{array}{c} p_{i}=\frac{\text{exp}(z_{i}/T)}{\sum_{j}\text{exp}\left(z_{j}/T\right)} \end{array}$$ (2)

where *T* controls the smoothness of the output distribution and *z*_*i*_ is the model’s predicted score for class i. To recover a standard softmax, *T* can be set to 1. The maximum probability over four politeness classes obtained through softmax gives the predicted polite level.

### 4.2 Generation model

We model **DG** and **G**, based on BART [34], following a standard seq2seq transformer based architecture. It comprises of a bidirectional encoder (like, BERT) and a left-to-right decoder (like, GPT). As in seq2seq transformers, each layer of BART’s decoder performs cross-attention over the final hidden layer of the encoder. To build our generation models, we fine-tune the pre-trained BART-large [34]. Due to the fact that BART comprises of an autoregressive decoder, it can be directly fine-tuned in the form of a sequence-to-sequence problem, where the input text corresponds to a user’s utterance (or agent’s utterance) and the output corresponds to an agent’s utterance (or candidate agent’s response). The model is trained in an end-to-end fashion, which trains the encoder to find an intermediate representaion between the output and input words by de-noising it. Using backpropagation of cross-entropy loss, the encoder is trained in two steps. First, only the randomly initialized source encoder, positional embeddings, self-attention input projection matrix are updated. Then in the second step, all the model parameters are trained.

### 4.3 Generative politeness adaptive dialogue system

In a task-oriented dialogue setting, to engage and request required information from a user, an agent should be able to form informative and adequate responses. Further, a user may loose interest in the ongoing dialogue due to redundant or generic agent’s responses. Therefore, a dialogue agent should be able to understand and learn user’s behaviour with the ongoing conversation itself and be able to generate interactive, empathetic and diverse responses, to adapt user’s needs concurrently. To incorporate all these aspects, we propose an effective end-to-end RL based generative politeness adaptive dialogue system (GenPADS), where at first utterance’s politeness semantics are extracted through a transformer-based politeness classifier. Second, a RL based dialogue agent uses this politeness information to design its reward feedbacks to adapt towards polite actions. Lastly, a transformer based generator model generates interactive and diverse responses.

Following the implementation [35], we chose the policy gradient approach [36] to design our reward functions. In traditional reward function, success of task is rewarded with positive constant, failure with 0 or negative constant and to complete the task sooner, it rewards -1 for each extra turn [25]. However, such a reward function neither takes into account any feedback information of the end user’s satisfaction nor it checks agent’s own actions for empathy and politeness, whereas it is natural for a human to plan his next action in a dialogue considering empathy and politeness levels of the conversational partner. Therefore, we propose a polite feedbacks based reward algorithm that checks user’s and agent’s utterances’ polite feedbacks to penalize dis-satisfactory actions and adapt polite actions. To get politeness feedbacks, we first use our politeness classifier (PC) to predict the politeness class of the user’s current action (*U*_*ca*_) and agent’s last action (*B*_*la*_) on-the-fly. Based on these predictions, we design immediate rewards for each turn for the RL based GenPADS. Due to the fact that GenPADS considers the user’s satisfaction and agent’s action quality information in the form of polite rewards, it may penalize its impolite actions receiving noisy user’s responses. This may force the system to form informative, polite and empathetic responses, hence completing the task-in-hand sooner compared to the traditionally used reward function. To check the politeness adaptation of GenPADS, we calculate politeness factor (*p*_*factor*_) in each of the dialogue as given below:

$$\begin{array}{c} p_{factor}=\frac{total\;number\;of\;agent^{\prime }s\;polite\;actions}{total\;number\;of\;agent^{\prime }s\;actions} \end{array}$$

In GenPADS, to form interactive and diverse responses, we incorporate a BART architecture based generation module **G**. It takes as input the retrieved action from the already defined actionID-to-template dictionary and outputs a diverse and contextually coherent response. An overview of the proposed GenPADS is depicted in algorithm 1. To compare GenPADS, we use a baseline retrieval based PADS (RetrievalPADS), which is same as GenPADS, without any generator module.

**Algorithm 1:** GenPADS

**1 Require:** *Trained domain specific Polite classifier **PC** and Generator **G***

**2 Ensure:**

 *1. max*_*steps* ≤ 30.

 *2. p* denotes politeness

 *3. total*_*pi*_ *denotes total_politeness_impoliteness*

 *4. p*_*factor*_ *denotes politeness factor in a dialogue*

**3** *U*_*ca*_ = *user*′*s*_*first*_*action*

**4** *p* = 0

**5** *total*_*pi*_ = 0

**6** **for** *i* ← 0 *max*_*steps* **do**

**7**  print U_*ca*_

**8**  *B*_*la*_ = *pg*_*reinforce*.*sampleAction*();

**9**  *agent*′*s*_*action* = *G*.*predict*(B_*la*_)

**10**  $PL_{B_{la}}=PC.predict({\text{B}}_{la})$

**11**  **if** $PL_{B_{la}}=2$ *or* 3 **then**

**12**   *total*_*pi*_ = *total*_*pi*_ + 1

**13**   *p* = *p* + 1

**14**  **else**

**15**   *total*_*pi*_ = *total*_*pi*_ + 1

**16**  print agent’s_action;

**17**  *U*_*ca*_ = *user*′*s*_*respond*_*to*_*B*_*la*_;

**18**  **if** *politeness based reward* **then**

**19**   *reward* = *R*_2_(*PRRP*)

**20**  **else**

**21**   *reward* = *R*_1_(*Baseline*)

**22** *p*_*factor*_ = *p*/*total*_*pi*_

Following [25, 37], we build a simulated generative politeness adaptive dialogue system (GenPADS), to test the effectiveness of proposed polite feedbacks based reward algorithm. For each of the seven domains in Taskmaster dataset, a single task is selected and simulated. In the simulated system, the agent requests different slot-values required as per the task in hand from a simulated user, such as: <round_trip_opt>, <departure> >< <arrival> and <dep_date> >< <dep_time> to search a flight in the flights domain. In response, the user informs the slot-values. Impossible actions such as providing information about an uncovered slot-value are prevented using a simple mask. Information about the task, number of slot-values, and number of actions for each domain are shown in Table 2.

**Table 2:** Statistics of user simulator for each of the seven domains.

Domain	Task	slot-values	actions
flights	flight_search	5	12
food-ordering	food_order	5	12
hotels	hotel_search	5	12
movies	movie_search	4	10
music	music_play	4	10
restaurant-search	restaurant_search	4	10
sports	sports_team_search	5	12

### 4.4 User simulator

A reinforcement learning system approximates its optimal policy by interacting with an environment. It continually rewards its chosen actions as per feedbacks received from the environment. Therefore, to train a reinforcement learner having a static dialogue corpus is not feasible. An optimal choice would be to run experiments with human subjects interacting with the RL based system which is unfortunately very expensive due to availability scarcity. Therefore, a common workaround in research community is to use user simulators mimicking the behaviour of real users in a consistent manner [38, 39]. We build seven user simulators focusing on a single task for each of the seven domains. Building of a user simulator depends on the creation of two essential components of a dialogue system *viz.* Dialogue Manager (DM)- ensuring the intrinsic logical consistency of user simulator, governing the simulator’s next action [40–42]; and Natural language generation (NLG)- controlling the extrinsic language fluency i.e. translating the semantic outputs from DM module to user understandable natural language [43, 44]. Our proposed work here builds an RL based DM module to adapt towards polite actions as per user’s dissatisfaction with the dialogue and a transformer based NLG module to generate diverse and interactive agent’s responses.

In Table 2, we give information about the task considered, number of slot-values for corresponding task and number of actions simulated for each of the seven domains. In a task-oriented dialogue system, a user simulator’s task is to achieve a pre-defined goal by interacting with the environment. Therefore, for Taskmaster dataset, we simulate a single goal for each of the seven domains. For example, in flight domain, goal of the user simulator can be flight search between the two stations. To simulate user for each of the domains, we follow a similar approach as used in [25, 37]. As an example, in flight domain, for each of the dialogue, the simulated user is initiated with a goal of providing information of five slot_values, then through the ongoing dialogue, provides slot_values placeholders, such as <round_trip_opt> as responses to agent’s information request actions. In a dialogue, users tend to give noisy responses, therefore, user’s responses were occasionally changed from inform “slot_value” to “noise” with probability = 0.10 for agent’s polite actions and with probability = 0.20 for agent’s impolite actions. Further, to penalize agent’s action we design two reward algorithms: one without any politeness feedback (a baseline) and other with politeness feedback (our proposed politeness based reward function).

#### 4.4.1 Baseline

To design the baseline reward as given in Algorithm 2, traditional reward function is chosen, i.e. we reward success of the task completion with 20, failure with -10 and penalization for each turn with -1 [25]. A task would be successful only if the user has provided all the requested slot-values for a given task, else it would be treated as failure.

**Algorithm 2:** Baseline Reward

**1:** **if** *success* **then**

**2:**  *R*_1_ = 20

**3:** **else if** *failure* **then**

**4:**  *R*_1_ = −10

**5:** **else**

**6:**  *R*_1_ = −1

#### 4.4.2 Politeness reward with repetition penalty (PRRP)

As stated earlier, users may tend to give noisy responses, therefore, our simulated user informs slot-values with probability *p* = 0.8 and *p* = 0.9 for agent’s impolite and polite actions, respectively. We expect that a user will provide more noisy response for an impolite response. This noisy response can be both an out-of-domain or error response. But, we need that agent should be able to penalize the impolite responses more as they tend to have more noisy responses showcasing user’s dissatisfaction with the dialogue. Besides, this may help the RL agent to converge towards success with better success rate. Based on the politeness feedbacks of *U*_*ca*_ and *B*_*la*_, we design our politeness based reward algorithm considering four immediate rewards {-2, -0.5, -1, -0.5} with the condition as shown in Algorithm 3.

Further, in an RL based simulated system, even if user has informed a slot-value for a agent’s action, it may take the same action repeatedly requesting the same slot. This, in effect, may degrade the quality of the ongoing dialogue and also dissatisfy the user. Therefore, we also penalize the repeated action in our proposed PRRP with reward -2.5. The detailed steps of PRRP are shown in Algorithm 3. We also perform the experiments with different reward choices with higher (such as {-3, -1, -2, -0.5}) and lower (such as {-1.5, -0.5, -1, 0}) reward set combinations. But, it is found that the success rate decreases with increments in penalization, whereas further decrease in penalization causes larger mean dialogue lengths. Our interpretation is that when increasing the penalization, the RL agent is not able to explore enough to converge, and when decreasing the penalization, the RL agent is not able to learn exploitation towards a single action.

**Algorithm 3:** PRRP Reward

**1 if** *success* **then**

**2**  *R*_2_ = 20

**3 else if** *failure* **then**

**4**  *R*_2_ = −10

**5 else if** *U*_*ca*_ = ‘*inform slot-value*’ **then**

**6**  $PL_{PL_{B_{la}}}=PC.predict\left({\text{PL}}_{B_{la}}\right)$

**7**  **if** *U*_*ca*_ = *N and* $PL_{B_{la}}=0\;or\;1$ **then**

**8**   *R*_2_ = −2

**9**  **else if** *U*_*ca*_ = *N and* $PL_{B_{la}}=2\;or\;3$ **then**

**10**   *R*_2_ = −0.5

**11**  **else if** *U*_*ca*_ ≠ *N and* $PL_{B_{la}}=0\;or\;1$ **then**

**12**   *R*_2_ = −1

**13**  **else if** *U*_*ca*_ ≠ *N and* $PL_{B_{la}}=2\;or\;3$ **then**

**14**   *R*_2_ = −0.5

**15** **else if** *repeated agent’s action* **then**

**16**  *R*_2_ = −2.5

**17 else**

**18**  *R*_2_ = −1

## 5 Experiments

### 5.1 Implementation details

The classification and generation experiments were performed using DistiBERT [33] and BART [34] based architectures, respectively. The polite classifier consist of 6 layers, 768 hidden units, 12 heads, 66 million parameters. Similarly, generation model consists of 24 layers, 1024 hidden units, 16 heads and 406 million parameters. All the classifiers are trained for 2 epochs and generators for 6 epochs. The hyper-parameters used to train the classifier and generation model are given below.

### 5.2 Hyperparameters details

We fine tune DistilBERT based polite classifier (PC) and BART based dialogue generator (DG) and generation module (G) considering different global (generally considered parameters values) and model (task-specific considered parameter values) parameters. The details of hyperprameter values are shown in Table 3.

**Table 3:** Hyperparameters information.

Polite classifier		Generation model DG and G	
Gobal	Local	Global	Local
*activation* = *gelu*	*adam*_*ϵ* = 1*e*−8	*activation* = *gelu*	*early*_*stopping* = *True*
*attention*_*dropout* = 0.1	*adafactor*_*clip*_*threshold* = 1.0	*attention*_*dropout* = 0.1	*length*_*penalty* = 2.0
*dim* = 768	*adafactor*_*decay*_*rate* = −0.8	*dim* = 1024	*max*_*length* = 128
*dropout* = 0.1	*dev*_*batch*_*size* = 8	*dropout* = 0.1	*number*_*return*_*sequences* = 1
*hidden*_*dim* = 3072	*learning*_*rate* = 4*e*−5	*hidden*_*dim* = 3072	*repetition*_*penalty* = 1.0
*max*_*position*_*embeddings* = 512	*train*_*batch*_*size* = 8	*max*_*position*_*embeddings* = 512	*eval*_*batch*_*size* = 4
*n*_*heads* = 12	*max*_*seq*_*length* = 128	*n*_*heads* = 16	*adam*_*epsilon* = 1*e*−8
*n*_*layers* = 6	*num*_*epochs* = 2	*n*_*layers* = 24	*num*_*epochs* = 10
-	*optimizer* = *AdamW*	*optimizer* = *AdamW*	-
-	-	*learning*_*rate* = 4*e*−5	-

### 5.3 Used Device configurations details

To train transformer based politeness classifier, generation models and RL frameworks GenPADS, we use following device configurations:

- **GPU:** NVIDIA GeForce RTX 2080 Ti.
- **Graphics Processor:** TU102.
- **Cores:** 4352.
- **TMUs:** 272.
- **ROPs:** 88.
- **Memory Size:** 11 GB.
- **Memory Type:** GDDR6.
- **Bus Width:** 352 bit.

### 5.4 Experimental setup

We implement GenPADS and Retrieval-PADS by considering both baseline and PRRP. To approximate optimal policy, policy gradient method is used as implemented in considering a discount factor of 0.9 following [19]. To build RL based dialogue system, we use HCN (hybrid code networks) [19] which combines an RNN with domain-specific knowledge encoded as dialogue system’s action templates. To optimize RL policy updates after each dialogue, we use LSTM with 32 hidden units and AdaDelta as optimizer for RNN architecture. Maximum number of turns allowed in a dialogue varied from 25 to 30 for each of the seven domains due to different number of slot-requesting actions in each of the domain. Following [45], *ϵ*-greedy exploration strategy is used for the RL agent. Lastly, in order to evaluate the performance of both the reward algorithms for all domains, the policy was frozen after every 400 episodes, and 500 simulated dialogues are run to compute the task success rate. We run the GenPADS and Retrieval-PADS for a total of 8000 episodes. All experiments are coded using Python language.

### 5.5 Automatic evaluation metrics

We evaluate polite classifier’s (PC) performance in terms of F1-score (F1) [46]. While the generation models *viz.* Dialogue Generator (DG) and GenPADS generation module (G) are evaluated using standard metrics, such as Perplexity (PP) [47], Bleu score (BLEU) [48], and NIST score (NIST) [49]. We evaluate RL frameworks, GenPADS and RetrievalPADS in terms of task completion rate (or success rate (SR)) and average number of turns needed to complete a task (or dialogue length (DL)), the two most widely used metrics to evaluate task oriented dialogue system [18, 25, 37, 50]. We use three more metrics to evaluate GenPADS and RetrievalPADS: (i). average politeness score (POL): used to judge agent’s adaptation towards polite actions; (ii). average meteor score (MET) [51] measuring the semantic similarity and (iii). average rogue-2 f-1 (R-2 F1) score [52]: measuring the diversity of the generated response.

### 5.6 Manual evaluation metrics

For human evaluation, we randomly sample 50 dialogues from the test set. We employ six annotators with post-graduate qualifications and having exposures in the similar task to evaluate the quality of the responses, generated by the different approaches in a similar manner as done in the literature [4, 8, 48]. First, we evaluate the quality of the response with respect to the four criteria: *Fluency* (F), *Informativeness* (I), *Politeness Adaptability* (PA) and *Diversity* (D). These are rated on a five-scale, where 1, 3, 5 indicate unacceptable, moderate, and excellent performance, respectively, while 2 and 4 are used for unsure. We compute Fleiss’ kappa to measure the inter-rater consistency. The Fleiss’ kappa for F, I, PA and D are 0.63, 0.68, 0.71 and 0.59, indicating moderate agreement.

## 6 Results and analysis

### 6.1 Automatic evaluation

Evaluation results of polite classifier **PC** and generation module **G** are shown in Table 4. It can be seen that our polite classifier achieves significant F1 score for all domains, hence, showcasing its efficacy to identify impolite/polite utterances. It can be pointed out that each of the domains consists of a different polite class distribution for its utterances, which in turn resulted into different politeness classifier function approximations. Hence, the evaluated metrics had different results for each of the seven domains in terms of F1 score. It can be seen that food-ordering domain achieves a significant F1-score of 0.96 as compared to other six domains. This can be due the two reasons: first, food-ordering utterances have less variation between them, second, polite class distribution is balanced between all utterances.

**Table 4:** GenPADS polite classifier (PC), generation module (G) evaluation results.

	PC	GenPADS Generation module G				
Domain	F1	PP	BLEU	NIST	MET	R-2 F1
Flights	0.92	1.912	0.052	0.186	0.641	0.472
Food-ordering	0.96	1.698	0.050	0.214	0.758	0.452
Hotels	0.94	1.972	0.065	0.207	0.664	0.504
Movies	0.95	2.137	0.039	0.1618	0.654	0.469
Music	0.93	2.367	0.037	0.133	0.555	0.379
Restaurant-search	0.95	2.156	0.047	0.162	0.669	0.494
Sports	0.92	1.762	0.018	0.069	0.739	0.585

It can also be observed that the generation module (G) shows good scores for PP, NIST, MET and R-2 F1 metrics and lower BLEU scores, signifying its capability to generate diverse responses without loosing semantics. It should be noted that for different domains, G-Dataset has 9540 to 2077 varying numbers of utterances. Therefore, seven different sequence-to-sequence trained models are obtained with respect to each of the seven domains. Hence, goodness of different approximations of generating function for each of the seven domains varied. This resulted in different performance metrics values in each of the domains. It can be seen that Perplexity (PP) score in sports domain is better as compared to all other domains showcasing that a better probability distribution function approximation of seq-to-seq utterances. Further, meteor (MET) score is also better for sports domain as compared to other six domains. Lastly, it can be inferred from R-2 F1 score of 0.585 of sports domain that the generated responses in this domain have most similar phrases as compared to other domains.

To analyse politeness adaptive behaviour of GenPADS, we plot politeness score with respect to the number of dialogues trained in all of the seven domains. Further, we also plot success rate depicting model’s task completion rate. The evaluation results of all three models *viz.* GenPADS, RetrievalPADS and Dialogue Generator are demonstrated in Table 5.

**Table 5:** Automatic evaluation results of GenPADS, RetrievalPADS and Dialogue Generator for all the domains with Baseline (BL) and proposed PRRP reward algorithms.

		GenPADS					RetrievalPADS					Dialogue Generator				
Domain	rew	DL	POL	MET	R-2 F1	SR	DL	POL	MET	R-2 F1	SR	PPL	BLEU	NIST	MET	R-2 F1
Flights	BL	10.8	0.587	0.627	0.511	0.67	11.1	0.674	0.999	0.999	0.67	6.18	0.038	0.132	0.127	0.059
	PRRP	**10.7**	**0.851**	0.721	0.543	**0.79**	**10.3**	**0.842**	0.999	0.999	**0.77**					
Food-ordering	BL	13.8	0.656	0.668	0.506	0.69	12.9	0.597	0.999	0.999	0.686	3.05	0.027	0.172	0.387	0.345
	PRRP	**11.5**	**0.936**	0.642	0.444	**0.86**	**12.6**	**0.908**	0.999	0.999	**0.84**					
Hotels	BL	10.9	0.849	0.665	0.462	0.74	12.8	0.804	0.999	0.999	0.71	7.15	0.087	0.261	0.146	0.078
	PRRP	**9.9**	**0.893**	0.709	0.573	**0.82**	**10.3**	**0.864**	0.999	0.999	**0.82**					
Movies	BL	9.9	0.744	0.701	0.446	0.77	11.8	0.694	0.999	0.999	0.74	7.45	0.015	0.058	0.146	0.086
	PRRP	**9.5**	**0.888**	0.700	0.407	**0.84**	**9.7**	**0.865**	0.999	0.999	**0.83**					
Music	BL	9.7	0.910	0.525	0.330	0.71	9.6	0.881	0.999	0.999	0.71	11.4	0.007	0.33	0.231	0.156
	PRRP	**9.4**	**0.959**	0.440	0.237	**0.86**	**9.4**	**0.921**	0.999	0.999	**0.84**					
Restaurant-search	BL	9.5	0.418	0.739	0.446	0.79	11.7	0.381	0.999	0.999	0.75	8.46	0.046	0.153	0.165	0.089
	PRRP	**8.5**	**0.920**	0.709	0.467	**0.82**	**9.7**	**0.940**	0.999	0.999	**0.78**					
Sports	BL	11.3	0.806	0.541	0.328	0.64	13.6	0.795	0.999	0.999	0.64	5.14	0.007	0.032	0.270	0.163
	PRRP	**10.9**	**0.948**	0.657	0.418	**0.81**	**11.5**	**0.915**	0.999	0.999	**0.79**					

GenPADS and RetrievalPADS were tested for 10,000 dialogues. Performance of the superior model is highlighted in bold.

**Fig 3:**
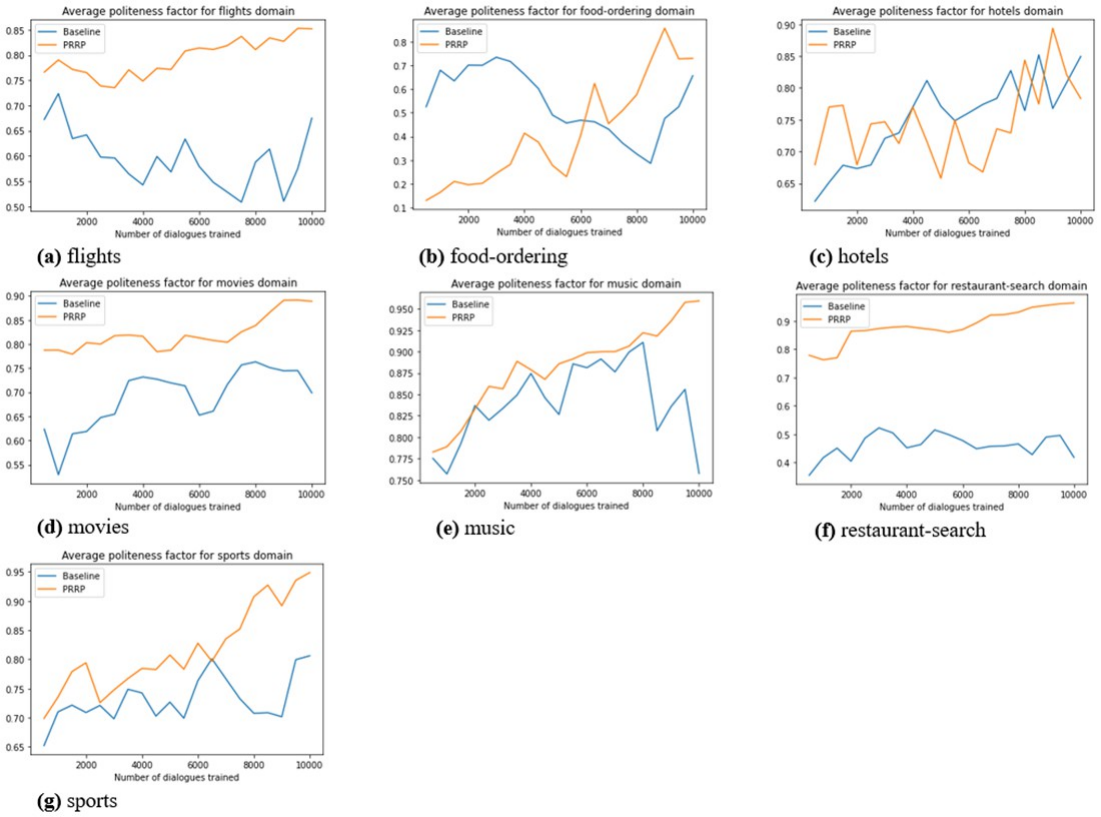
Average politeness factor with different reward algorithms for (a) flights (b) food-ordering (c) hotels (d) movies and (e) music (f) restaurant-search (g) sports domain.

**Fig 4:**
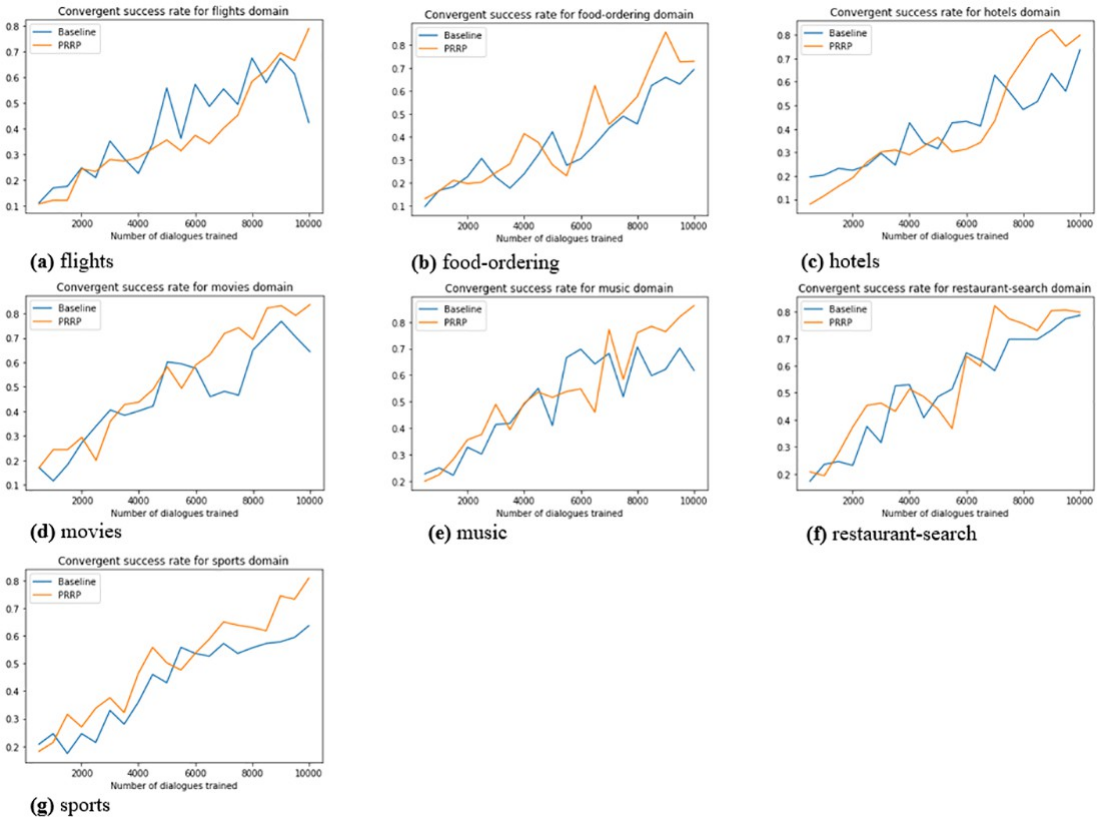
Average convergent success rate with different reward algorithms for (a) flights (b) food-ordering (c) hotels (d) movies and (e) music (f) restaurant-search (g) sports domain.

From Fig 3, it is evident that politeness factor for each domain shows a consistent increase with the number of dialogues trained. It can be seen that PRRP directs the RL agent more towards polite actions as compared to the baseline. This is due to the fact that PRRP gives hard penalization to agent’s impolite and noisy slot-value receiving responses. In terms of task success rate, it can be inferred from Fig 4 that PRRP performs better than the baseline—showing consistent convergence towards task completion. It is due to the fact that, Baseline uses same penalization for each type of action, assigning same priority to all actions whereas, PRRP penalizes the impolite and noisy slot receiving actions, hence, the probability of getting slot-value information becomes higher for PRRP. In flight, restaurant search and movies domains consistent increase in politeness factor can be seen. It can be due to the fact that polite actions are less penalized than the others. In food-ordering, music and sports domains, due to absence of polite reward and hard penalization, PRRP drives the agent towards more polite action than the Baseline. In hotel domain, the adaptation towards polite actions and retaining it is getting difficult as Baseline model also is performing near to similar to PRRP, hence, due to unclear differentiation between these two algorithms it is hard to decide which algorithm is more politeness-adaptive. But, considering PRRP clearly adapts more towards polite actions in other six domains, it can be inferred that PRRP is better than the Baseline to force the agent toward polite actions.

Fig 4 shows the average convergent success rate with both Baseline and prposed PRRP. It is evident from the figure that PRRP outperforms the Baseline in all seven domains. Additionally, it can be seen that when compared to the baseline, PRRP consistently converge towards success. It may be because baseline investigates all acts, hence converges towards success only when it investigates polite actions in some episodes only; yet, when it encounters the rude behaviours, the likelihood of receiving noisy responses increases, leading to divergence from success. Therefore, it can be claimed that prposed PRRP will adjust an RL agent more towards a consistent success rate than the baseline once they have been run for a significant number of episodes.

GenPADS and RetreivalPADS are designed in the same RL frameworks only with the difference that GenPADS uses a generation module to generate agent’s responses, whereas RetreivalPADS retrieves the action from a action-ID template based dictionary. Therefore, it can be seen from Table 5 that MET and R-2 F1 scores of RetreivalPADS is approximately 1 with no major change as it always retrieves the same action. It can also be inferred from MET and R-2 F1 scores of GenPADS that it is able to generate semantically same but diverse responses. High MET scores depicts the repetitive generation of the same word(s) in the output responses, whereas mid-low scores of R-2 F1 depict that the generated response is diverse from the input template based response. It can also be observed from Table 5 that for all the domains, polite based reward algorithm, PRRP, in terms of success rate (SR) and dialogue length (DL), performs better than the baseline. In most cases, it can be seen that the proposed PRRP is able to complete dialogues with shorter lengths than existing Baseline. This is likely due to PRRP’s emphasis on strongly penalising impolite behaviours with the repetitions in the dialogue. Further, it can also be observed that there is minor difference in terms of success rate (SR) and dialogue length (DL) between GenPADS and RetrievalPADS. This might be due to the use of similar rewards because of the same RL frameworks. It can be deduced that in each domain, providing the agent with polite feedback from both the user and the agent improved the system’s success rate compared to the baseline.

From Table 5, it can be inferred from the evaluation of different metrics of dialogue generator that the generated responses vary significantly from the real agent’s responses, depicting the information loss due to the absence of a DM module, which keeps a track of its dialogue states showing slot-value information it received. Further, MET and PP scores of Dialogue generator module depict that it is able to generate meaningful sentence. Due to absence of DM module, DG is not evaluated in terms of SR, DL and POL metrics.

### 6.1 Human evaluation

In Table 6, we present the results of human evaluation for the baseline and the proposed framework for the GenPADS framework. Here, we provide the average results for all the domains. As it is evident from the table, the fluency of the PRRP framework is better than the Baseline network indicating that the dialogues are grammatically correct and fluent. Similarly, the loss of information is important to consider while incorporating politeness, therefore it is visible that the *informativeness* metric score is higher for *PRRP* framework. This ensures that there is no loss of information in the responses. As the primary contribution is to infuse politeness, hence on manual evaluation, we see that the politeness adaptability scores are superior compared to the baseline. Finally, it is significant to have diverse responses to increase the inter-activeness and avoid generic replies. From the table, the diverse scores are also higher in comparison to the baseline framework. Therefore, it can be concluded that the responses are fluent, polite, diverse and informative.

**Table 6:** Results of manual evaluation.

Domains	F	I	PA	D
***Baseline***	3.87	3.32	3.18	3.54
***PRRP***	**4.16**	**3.87**	**4.08**	**3.91**

### 6.2 Error analysis

Our proposed model with PRRP reward function may show same behaviour as compared Baseline reward function. If Baseline initialises with a polite action and randomly each and every time selects only polite action, then it will show similar behaviour with respect to PRRP, as is evident from Fig 3(c), where Baseline reward function also nearly shows same politeness adaptation behaviour as PRRP. This in turn also affects the success rate of the proposed reward function PRRP as is evident from Fig 4(f) that baseline reward function may show similar performance in comparison to PRRP due to less penalization of impolite actions. But, it can also be observed that after 6000 dialogues trained, PRRP consistently shows better performance than the Baseline reward function. It could be due the repetition penalty present in the PRRP reward function. It is also seen that sometimes for both Baseline and PRRP reward functions MET score and R-2 F1 varied. It is due to the fact that here, generation module **G** can generate semantically similar but diverse response each time for a selected response, which may result in different scores of MET and R-2 F1. Our main aim is to adapt politeness in an ongoing dialogue using a well designed reward function and to have diversity in generated responses such that dialogue may become more interactive, which as per results is well achieved by proposed system **GenPADS**.

## 7 Discussion

**Fig 5:**
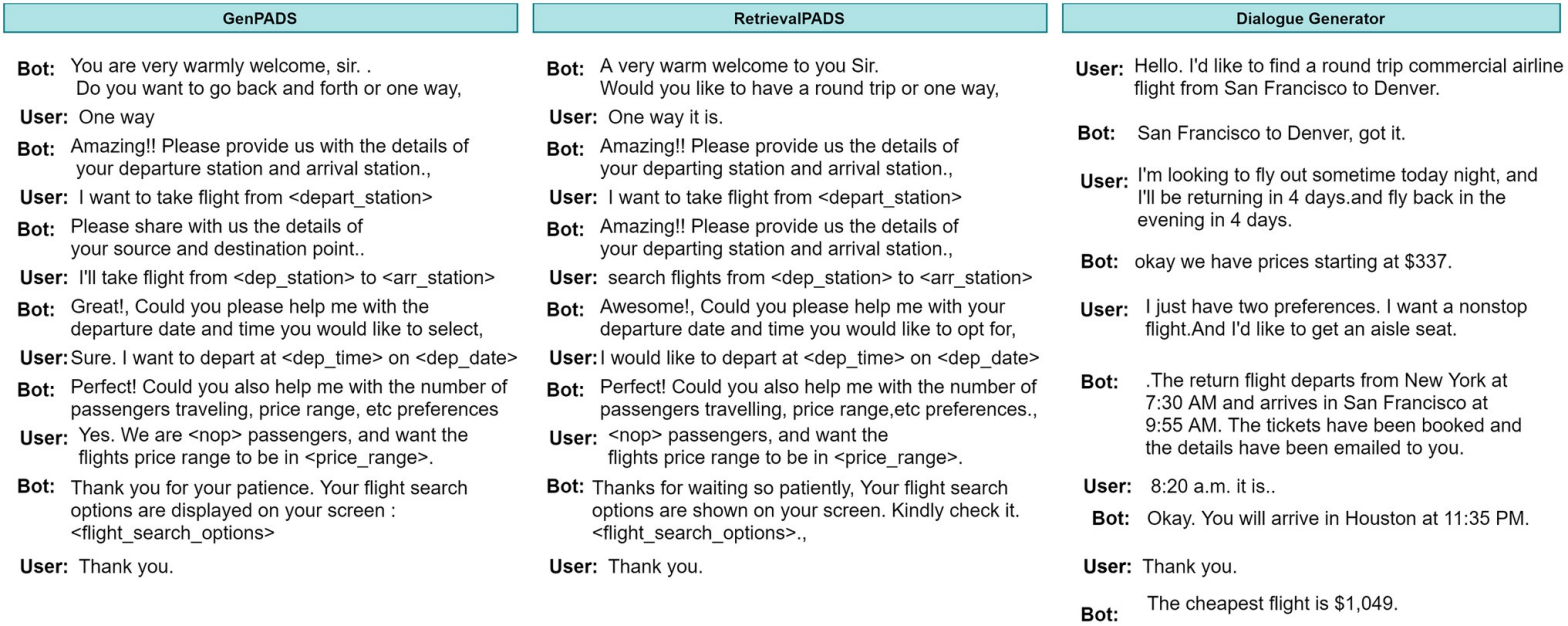
A conversation example of flight search using GenPADS, RetrievalPADS and Dialogue Generator.

To compare GenPADS, RetrievalPADS and Dialogue generator (DG), we show in Fig 5 a flight search dialogue example adopted from all these three models. It can be observed from the figure that DG generates sometimes random responses such as: *‘The return flight departs from New York at 7:30 AM and arrives in San Francisco at 9:55 AM. The tickets have been booked and the details have been emailed to you.’*, which may be relevant to the utterance, but is inconsistent to the context of the ongoing dialogue, hence causing information loss. It can also be seen that GenPADS and RetrievalPADS nearly perform similarly with only the difference that GenPADS generates agent’s utterance using the generation module (G), which causes it to generate a diverse action when requesting again for departure and arrival station compared to the RetrievalPADS which requests again with same action. For example: to ask source and destination point again, RetrievalPADS says *’Amazing! Please provide us the details of your departing and arrival station’*—the same response as previously asked whereas GenPADS says *‘Please, share us with the details of your source and destination point’*—a semantically similar but diverse response from the previous one. This helps GenPADS in achieving interactiveness in its responses, hence, ensuring better engagement.

## 8 Limitations

For different task-oriented datasets, as per different actions and slots values available for each of the task a different user simulator can be designed. After having, different user simulator, our proposed system can be used for any of the task-oriented dataset effectively. Designing of user simulators can be a cumbersome task for designers which limits the system. Second, at the first hand the designed responses are template based in a well defined user simulator. This user simulator cannot totally ensure the real time user’s behaviour. At real time, same user’s tend to express different attitudes at different times which is a very challenging task. Therefore, this user simulator ensures only an approximated well defined user’s behaviour. To achieve, a pure real time user’s behaviour simulation, further research is needed which opens up the door for our future research works.

## 9 Conclusion and future work

To ensure customer satisfaction and increase customer retention it is crucial to incorporate politeness and diversity in a dialogue agent’s responses. Focusing on these aspects, in this paper, we have proposed a novel task of developing a generative politeness adaptive dialogue system. To the best of our knowledge, this is the very first attempt that focuses on incorporating all three aspects informativeness, politeness and diversity in an end-to-end RL based learning framework. Firstly, we prepare two novel datasets, PADD and GenDD using Taskmaster-2 dataset. We used these datasets to build a politeness classifier **PC** and a response generation model **G**. Then, these models are incorporated in an RL based framework, GenPADS by designing a politeness based reward function. The proposed reward function receives politeness information from the **PC** as rewards. If negative reward is obtained as feedback, the proposed RL-based system adapts to satisfy the user by taking polite actions. Then, the selected action from the dialogue agent is passed through **G** to generate interactive diverse responses. Experimental analysis shows that by incorporating politeness based rewards in the conversation, as and when required facilitates the GenPADS to achieve a better success rate and shorter dialogue length. Further, generation module in GenPADS makes the dialogue interactive and engaging.

In the future, we would like to extend it for multiple intents to optimize politeness in dialogues for the agent efficiently. Further, our proposed system simulates one domain at a time, but we would also like to work on a system which can simulate multiple domains at same time.

## Supporting information

S1 Data (ZIP)
